# Statin administration or blocking PCSK9 alleviates airway hyperresponsiveness and lung fibrosis in high-fat diet-induced obese mice

**DOI:** 10.1186/s12931-024-02842-x

**Published:** 2024-05-18

**Authors:** Lin Liang, Sook In Chung, Tae-Eun Guon, Kyung Hee Park, Jae-Hyun Lee, Jung-Won Park

**Affiliations:** 1 Graduate School of Medicine, Yonsei University College of Medicine, Seoul, Korea; 2 Institute of Allergy, Yonsei University College of Medicine, Seoul, Korea; 3 Division of Allergy and Immunology, Department of Internal Medicine, Yonsei University College of Medicine, 50-1 Yonsei-ro, Seodaemun-gu, Seoul, 03722 Korea

**Keywords:** Obesity, Statin, PCSK9, Asthma, Alirocumab

## Abstract

**Background:**

Obesity is associated with airway hyperresponsiveness and lung fibrosis, which may reduce the effectiveness of standard asthma treatment in individuals suffering from both conditions. Statins and proprotein convertase subtilisin/kexin-9 inhibitors not only reduce serum cholesterol, free fatty acids but also diminish renin-angiotensin system activity and exhibit anti-inflammatory effects. These mechanisms may play a role in mitigating lung pathologies associated with obesity.

**Methods:**

Male C57BL/6 mice were induced to develop obesity through high-fat diet for 16 weeks. Conditional TGF-β1 transgenic mice were fed a normal diet. These mice were given either atorvastatin or proprotein convertase subtilisin/kexin-9 inhibitor (alirocumab), and the impact on airway hyperresponsiveness and lung pathologies was assessed.

**Results:**

High-fat diet-induced obesity enhanced airway hyperresponsiveness, lung fibrosis, macrophages in bronchoalveolar lavage fluid, and pro-inflammatory mediators in the lung. These lipid-lowering agents attenuated airway hyperresponsiveness, macrophages in BALF, lung fibrosis, serum leptin, free fatty acids, TGF-β1, IL-1β, IL-6, and IL-17a in the lung. Furthermore, the increased RAS, NLRP3 inflammasome, and cholecystokinin in lung tissue of obese mice were reduced with statin or alirocumab. These agents also suppressed the pro-inflammatory immune responses and lung fibrosis in TGF-β1 over-expressed transgenic mice with normal diet.

**Conclusions:**

Lipid-lowering treatment has the potential to alleviate obesity-induced airway hyperresponsiveness and lung fibrosis by inhibiting the NLRP3 inflammasome, RAS and cholecystokinin activity.

**Graphical abstract:**

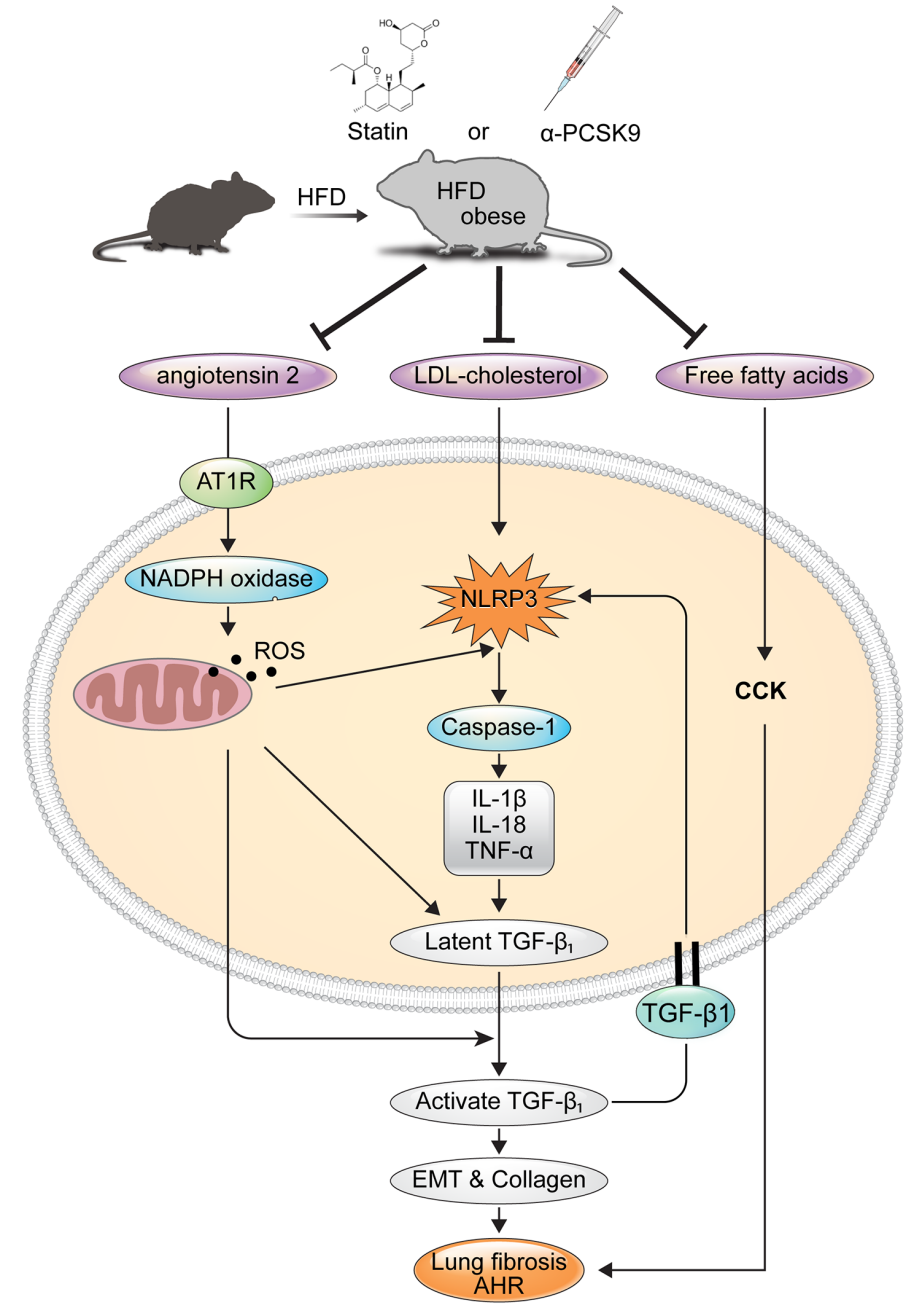

## Introduction

Obesity correlates with metabolic dysfunction and exacerbates asthma incidence and severity [1]. According to the Global Initiative for Asthma 2022, asthma with obesity is now recognized as a distinct phenotype, often associated with heightened symptoms, resistance to corticosteroids, and non-eosinophilic airway inflammation [2].

Animal studies highlight that a high-fat diet (HFD) induces obesity in mice, resulting in airway hyperresponsiveness (AHR) and pulmonary fibrosis [3]. Elevated systemic pro-inflammatory markers like C-reactive protein, TNF-α, TGF-β, leptin, and IL-6 are linked to the obesity [4].

The NLRP3 inflammasome pathway, activated by metabolic damage-related factors such as excessive glucose, reactive oxygen species (ROS), oxidized lipids, and cholesterol crystals, contributes to inflammatory responses, insulin resistance, and metabolic syndrome [5]. In obesity-related asthma, heightened oxidative stress and NLRP3 inflammasome activation in airways are implicated in symptom manifestation [6, 7]. Increased renin and angiotensin II levels in obese mice suggest a potential link between the renin-angiotensin system (RAS) and fibrosis development in various organs, including the lungs [8, 9]. Cross-talk between RAS and TGF-β1 signaling pathways, facilitated by ROS-NLRP3 pathway, contributes to fibrosis [10]. Consequently, inhibiting RAS activation, NLRP3, and TGF-β1 expression could offer novel therapeutic avenues for managing asthma in patients with both obesity and asthma.

Furthermore, in obese mice, elevated free fatty acid (FFA) levels stimulate cholecystokinin (CCK) secretion, potentially contributing to AHR via enhanced CCK receptor expression in lung smooth muscles. Blocking CCK receptors has shown promise in mitigating AHR in asthma with obesity patients [11].

Statins and PCSK9 inhibitors, known for lowering LDL-cholesterol, exhibit anti-inflammatory effects. PCSK9 binds to LDL receptor on cell surface and induce intracellular degradation of LDL receptors, and cause hypercholesterolemia. Subsequently, human monoclonal antibodies that target PCSK-9 decrease LDL cholesterol level [12]. PCSK9’s role in LDL receptor degradation correlates with heightened PCSK9 levels in obesity [13, 14], while TGF-β1 induces PCSK9 secretion [15]. Studies suggest PCSK9 knockout suppresses NLRP3 inflammatory pathways [16], and statins exhibit inhibitory effects on NLRP3 and TLR signaling pathways, potentially beneficial for airway inflammatory diseases [17–20].

This study aims to assess the impact of statins or PCSK9 inhibitors on AHR and lung fibrosis in an HFD-induced obesity model of mouse. It seeks to unravel mechanisms of these lipid lowering agents involving RAS activation, NLRP3 inflammasome, pro-inflammatory cytokines, and CCK expression.

## Materials and methods

### Study scheme and animals

**Fig. 1:**
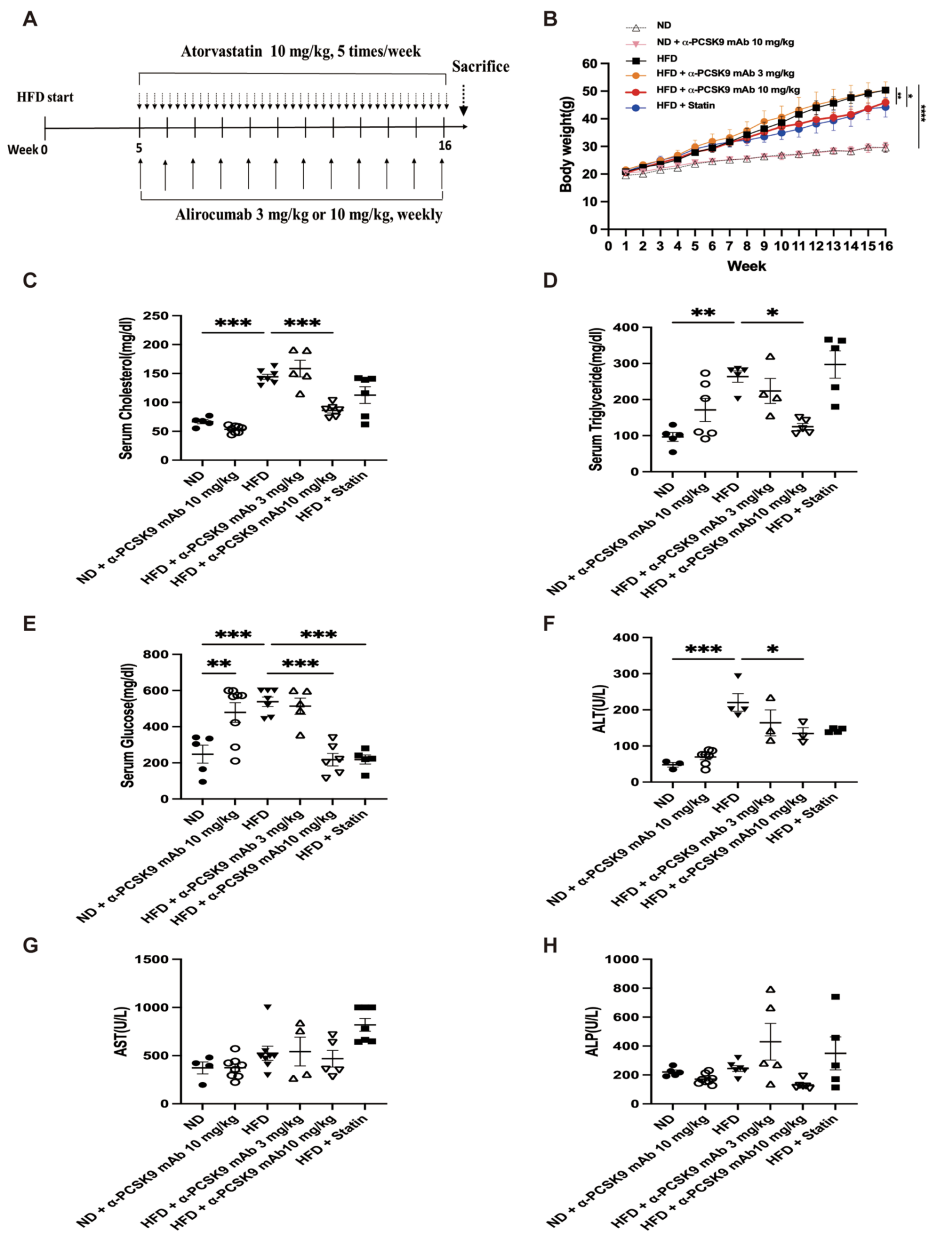
Effects of PCSK9 inhibition or statin administration on body weight and serum biochemical marker levels in HFD-induced obese mice. (**A**) Experimental scheme of PCSK9 inhibition or statin treatment in HFD or normal chow-fed mice. (**B**) Body weight in mice on the ND or HFD. (**C**) Cholesterol, (**D**) triglyceride, (**E**) glucose, (**F**) Alanine aminotransferase (ALT), (**G**) Aspartate aminotransferase (AST), and (**H**) Alkaline phosphatase (ALP) levels were measured in serum. The results are expressed as the mean ± SEM (*n* = 6 per group). Statistical analysis of body weight changes was performed using repeated-measures ANOVA, and other analyses were performed with one-way ANOVA with Bonferroni correction. *: *P* < 0.05, **: *P* < 0.01, and ***: *P* < 0.001. ND: normal diet; HFD: high-fat diet

Male C57BL/6 mice were fed a normal diet (ND) or HFD for 16 weeks. The diets consisted of an isocaloric control diet (fat comprised 10% of calories; D12450; Research Diets Inc.) or an HFD (fat comprised 60% of calories; D12492; Research Diets Inc.). The mice were weighed every week. Administration of the anti-PCSK9 monoclonal antibody alirocumab (Biorbyt, Cambridge, UK) or atorvastatin (Cayman Chemical, Ann Arbor, MI, USA) began at week 5. Alirocumab was injected (3 or 10 mg/kg) weekly for 16 weeks. Atorvastatin was orally administered five times per week for the same 16-week period at a dose of 10 mg/kg (Fig. 1A). Lee and colleagues kindly provided the triple-transgenic TGF-β1 mice for this study [21]. Male and female transgene^+^ mice, and transgene^−^ littermates aged 6–8 weeks were fed 0.5 mg/ml doxycycline in water ad libitum for 4 weeks with intraperitoneal injection of alirocumab (10 mg/kg) weekly and oral administration of atorvastatin (10 mg/kg) five times per week (Fig. 8A). All animal procedures were performed according to the Institutional Animal Care and Use Committee (IACUC) regulations of Yonsei University College of Medicine (Seoul, Korea), which has been fully accredited by the Association for Assessment and Accreditation of Laboratory Animal Care International (IACUC approval number: 2021 − 0256).

### In vitro Bronchial Epithelial Cell Stimulation with TGF-β1

Human bronchial epithelial BEAS-2B cells were grown and maintained in bronchoepithelial basal medium (Lonza, Basel, Swiss) with supplements in 6-well plates. Following a 24-hour serum starvation, cells were treated with alirocumab (10 µg/ml) or atorvastatin (20 µM) for 24 h and then stimulated with TGF-β1 (10 ng/ml) for 10 min. Real-time PCR analysis was conducted.

### Measurement of AHR

AHR was assessed using the FlexiVent system (SCIREQ, Montreal, QC, Canada). To determine the baseline airway resistance (Rrs), mice were exposed to nebulized phosphate-buffered saline for 3 min followed by progressive exposure to 6.25, 12.5, 25, 50, and 100 mg/ml nebulized methacholine (MCh; Sigma-Aldrich, St. Louis, MO, USA) using an ultrasonic nebulizer (DeVilbiss, Somerset, PA, USA). Each test lasted for 3 min, and average Rrs values were calculated for each MCh concentration.

### Bronchoalveolar lavage

Prior to tracheostomy, mice were anesthetized by intraperitoneal injection of pentobarbital (50 mg/kg; Hanlim Pharma Co., Seoul, Korea). A 23-gauge needle was used to insert a silicone tube into the mouse trachea, which was connected to an 800-µl tuberculin injector to deliver 1 ml of Hank’s balanced salt solution (HBSS; Thermo Fisher Scientific, Waltham, MA, USA) to the lungs. The recovered bronchoalveolar lavage fluid (BALF) was centrifuged for 3 min at 10,000 rpm at 4 °C, and the supernatant was stored at − 70 °C. Whole cells were re-suspended in HBSS, and BALF cell smears were prepared using cytocentrifugation (Thermo Shandon Cytospin 3, Marshall Scientific, Hampton, NH, USA) and then stained with Diff-Quick (Sysmax, Kobe, Japan). The percentages of macrophages, eosinophils, lymphocytes, and neutrophils in BALF were determined by counting 500 leukocytes in randomly selected fields under a light microscope.

### Serum biochemical assays

Serum was harvested following centrifugation of clotted blood samples and examined for the following biochemical parameters using an automated clinical chemistry analyzer (Dri-Chem 4000i, Fujifilm, Japan): serum cholesterol, triglyceride, glucose, alanine aminotransferase (ALT), aspartate aminotransferase (AST), and alkaline phosphatase (ALP).

### Enzyme-linked immunosorbent assays (ELISAs)

To analyze cytokine levels, 100 mg of right lung tissue was lysed using a tissue homogenizer (Biospec Products, Bartlesville, OK, USA) with RIPA buffer (Thermo Fisher Scientific Inc., Rockford, IL, USA). After incubation on ice for 30 min, the homogenates were centrifuged at 10,000 rpm for 10 min. Lung homogenate supernatants were collected, passed through a 0.45-µm filter (Gelman Science, Ann Arbor, MI, USA), and stored at − 80℃ to measure cytokines, angiotensin II, and angiotensin II receptor type 1 levels. The measured cytokine levels were adjusted to the lung tissue weight. IL-1β, IL-6, IL-17a, TGF-β1, TNF-α, and leptin concentrations in lung homogenate or serum were measured using commercial ELISA kits (R&D Systems, Inc. Minneapolis, MN, USA).

To evaluate the degree of oxidative stress, the concentration of malondialdehyde (MDA) in lung homogenate or serum was measured using an ELISA (DoGenBio, Seoul, Korea). CCK was detected in lung homogenate or serum using the CCK Enzyme Immunoassay Kit (Biorbyt). An FFA Assay Kit (DoGenBio) was used to detect FFAs in lung homogenate or serum. The procedures were performed following the recommended manufacturer’s protocols.

### RNA purification, reverse transcription, and real-time PCR amplification

Total RNA was isolated from extracted lungs using the TRIzol reagent (Invitrogen, Carlsbad, CA, USA). cDNA was then synthesized using an RNA to cDNA EcoDry premix kit (Takara Bio, Kusatsu, Japan) following the manufacturer’s recommended protocol. PCR master mix (Power SYBR Green PCR Master Mix, Applied Biosystems, Warrington, UK) was used to perform quantitative RT-PCR with a StepOnePlus^™^ PCR System (Applied Biosystems). The relative expression levels of target genes were normalized to the β-actin expression levels. The primer sequences are shown in Table 1.

**Table 1:** Sequences of quantitative RT-PCR primers

Genes	Forward primers sequence (5’→3’)	Reverse primers sequence (5’→3’)
Collagen 1_Ms	TGGGATTCCCTGGACCTAA	GCTCCAGCTTCTCCATCTTT
Collagen 3_Ms	ATCTGAGGGCTCGCCCGGT	CAATGGCAGCACCGCCACCA
Fibronectin_Ms	TCAGAAGAGTGAGCCCCTGA	GGAAGGGTAACCAGTTGGGG
NLRP3_Ms	GCTGCTGAAGATGACGAGTG	TTTCTCGGGCGGGTAATCTT
Caspase-1_Ms	TCATTTCCGCGGTTGAATCC	CCAACAGGGCGTGAATACAG
ASC_Ms	ATGCCAACCAAAGCCAGAAG	CCTTGGGGTTGGAGAGATGA
IL-1β_Ms	GGCTCATCTGGGATCCTCTC	TCATCTTTTGGGGTCCGTCA
β-actin_Ms	CGCCACCAGTTCGCCATGGA	TACAGCCCGGGGAGCATCGT
GAPDH_Hm	CACATCGCTCAGACACCATG	TGACGGTGCCATGGAATTTG
NLRP3_Hm	TCTCATGCTGCCTGTTCTCA	CAAGGAGATGTCGAAGCAGC
Caspase-1_Hm	CCGAGCTTTGATTGACTCCG	TTCTGAGCCTGAGGATGTGG
IL-1β_Hm	GGAGAATGACCTGAGCACCT	GGAGGTGGAGAGCTTTCAGT

(Ms: mouse, Hm: human)

### Histopathology and immunohistochemistry

The left lung was perfused with 4% paraformaldehyde solution and then embedded in paraffin. Hematoxylin and eosin (H&E) staining was performed on lung sections to evaluate tissue inflammation, and periodic acid–Schiff (PAS) staining was employed to detect goblet cell hyperplasia and submucosal gland hypertrophy. In addition, Masson’s trichrome (MT) staining was conducted to assess fibrosis.

For NLRP3 and caspase-1 immunohistochemical staining (IHS), lung sections from each paraffin block were deparaffinized with xylene and rehydrated in ethanol. Antigen retrieval was conducted by autoclaving the sections at 120 °C for 15 min in citrate buffer (pH 6.0). Then, the sections were incubated in 3% hydrogen peroxide for 15 min to inactivate endogenous catalase. Next, the sections were incubated with anti-NLRP3 (1:100, SAB, USA), anti-caspase-1 (1:200, Abcam, Cambridge, UK), and anti-alpha smooth muscle actin (α-SMA) (1:100, Abcam) antibodies overnight at 4 °C. Finally, the sections were incubated with streptavidin horseradish peroxidase, and the percentage of positively-stained area was calculated using ImageJ software.

### Statistical analysis

Data were analyzed using Prism software (GraphPad Inc., San Diego, CA, USA). Changes in AHR and body weight were evaluated by repeated-measures analysis of variance (ANOVA) and a Bonferroni post-hoc test, and differences between the other variables were compared by one-way ANOVA and a Bonferroni post-hoc test. A value of *P* < 0.05 was considered statistically significant.

## Results

### PCSK9 inhibition or statin administration induced weight loss and altered serum biochemical marker levels in HFD-induced obese mice

The HFD resulted in significant weight gain compared with the standard chow diet (Fig. 1B). Among the HFD groups, administration of 10 mg/kg alirocumab (*P* = 0.005) or statin (*P* = 0.024) significantly reduced body weight, but 3 mg/kg alirocumab did not exhibit the same effect. The HFD notably influenced serum cholesterol (*P* < 0.001), triglyceride (*P* = 0.002), glucose (*P* < 0.001), and ALT (*P* < 0.001) levels. Treatment with alirocumab at 10 mg/kg reduced these levels to those observed in the ND group, whereas 3 mg/kg alirocumab did not yield a similar effect (Fig. 1C–H). However, in normal diet (ND)-fed mice, alirocumab treatment resulted in increased serum glucose levels (*P* = 0.009). Additionally, the statin displayed a trend in reducing blood lipid levels and demonstrated a statistically significant decrease in serum glucose level (*P* < 0.001).

### PCSK9 inhibition or statin administration suppressed AHR, monocytosis in BALF, and systemic pro-inflammatory mediators in the lungs of HFD mice

**Fig. 2:**
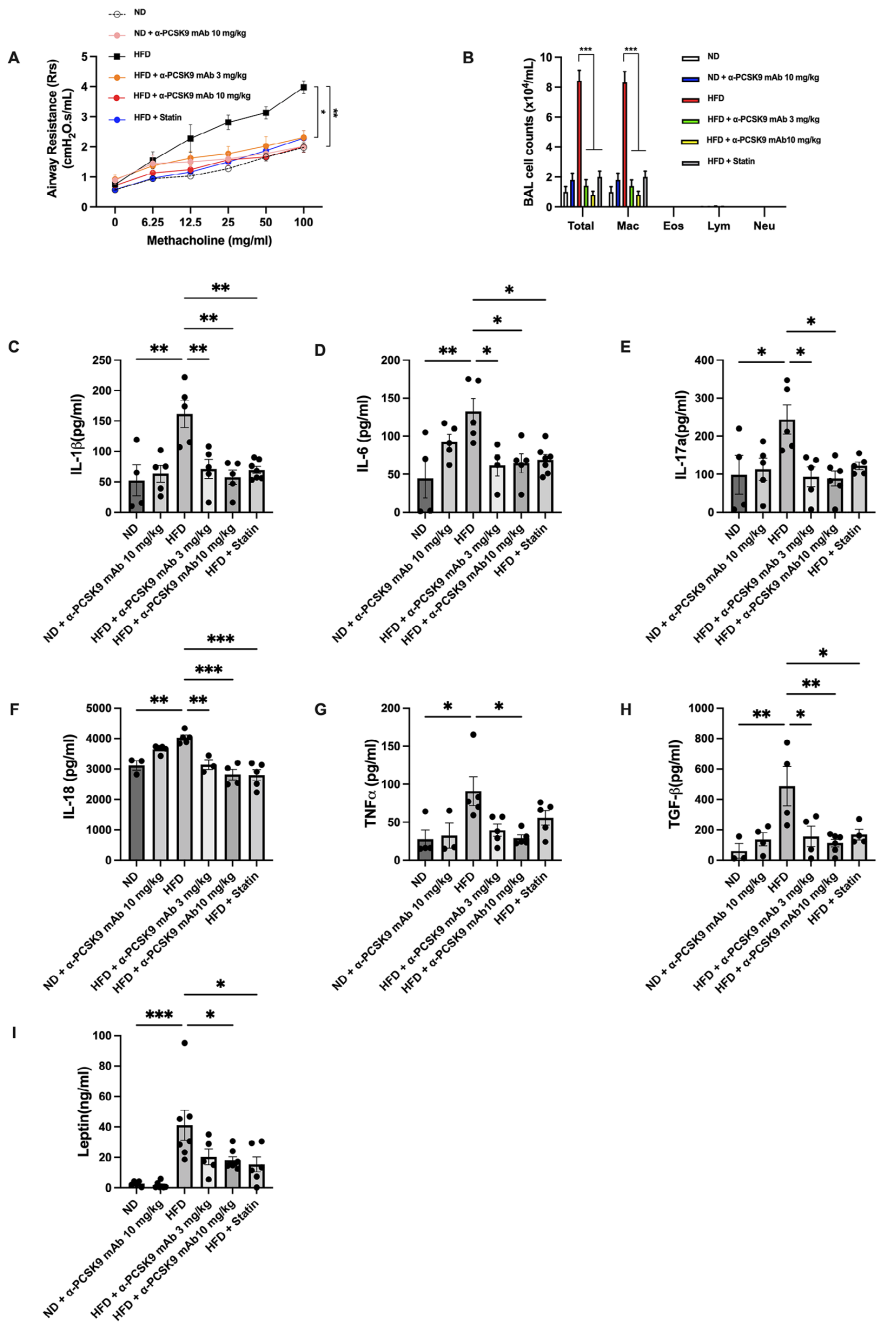
Effects of PCSK9 inhibition or statin administration on AHR and airway inflammation in HFD-induced obese mice. (**A**) AHR was measured as the Rrs at 24 h after the final treatment. (**B**) Effect of the HFD on cell counts in BALF. Mice were sacrificed 24 h after the final treatment, and BALF cells were isolated. (**C**–**H**) IL-1β (**C**), IL-6 (**D**), IL-17a (**E**), IL-18 (**F**), TNF-α (**G**), and TGF-β (**H**) levels in lung homogenates and leptin (**I**) levels in serum were evaluated by ELISAs. The results are expressed as the mean ± SEM (*n* = 6 per group). Statistical analysis of AHR was performed using repeated-measures ANOVA, and other analyses were performed using one-way ANOVA with Bonferroni correction. *: *P* < 0.05, **: *P* < 0.01, and ***: *P* < 0.001

The HFD group exhibited significantly increased AHR compared to the ND group (*P* = 0.005). Treatment with either alirocumab (3 mg/kg: *P* = 0.018 and 10 mg/kg: *P* = 0.003) or the statin (*P* = 0.022) alleviated AHR in the HFD group (Fig. 2A). BALF analysis revealed higher total cell number (*P* < 0.001) and macrophage count (*P* < 0.001) in the HFD compared to the ND group, with no notable increase in eosinophils, neutrophils, and lymphocytes due to the HFD. PCSK9 inhibition or statin administration reduced the total cell and macrophage counts in the HFD group (Fig. 2B).

The lungs of HFD mice showed significantly increased expression of IL-1β, IL-6, IL-17a, IL-18, TGF-β, and TNF-α, along with elevated serum leptin compared to the ND group. Alirocumab (10 mg/kg) notably attenuated these pro-inflammatory cytokines (Fig. 2C–I). Statin administration also attenuated these parameters, except for IL-17a and TNF-α.

### PCSK9 inhibition or statin administration attenuated fibrosis and epithelial-mesenchymal transition (EMT) markers in HFD mouse lungs

**Fig. 3:**
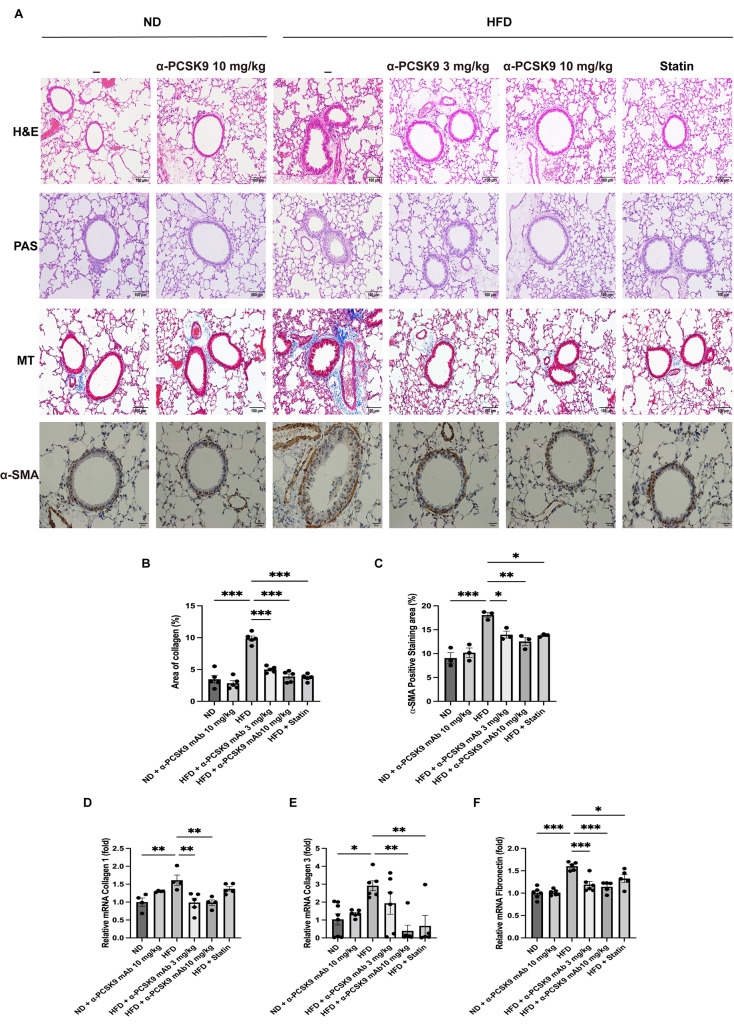
Effects of PCSK9 inhibition or statin administration on fibrosis and EMT markers in HFD mouse lungs. (**A**) Paraffin-embedded lung tissue sections underwent H&E, PAS, MT (original magnification: 100×), and α-SMA IHS (original magnification: 50×) staining. (**B-C**) Quantitative analyses of the fibrosis and peribronchial α-SMA-positive staining areas were performed using an image analysis system. Quantification of EMT markers in the lungs was performed using mRNA expression of collagen 1 (**D**), collagen 3 (**E**), and fibronectin (**F**). The results are expressed as the mean ± SEM (*n* = 6 per group). Statistical analysis was performed using one-way ANOVA with Bonferroni correction. *: *P* < 0.05, **: *P* < 0.01, and ***: *P* < 0.001. EMT: epithelial–mesenchymal transition, H&E: hematoxylin and eosin; IHS: immunohistochemical staining; MT: Masson-trichrome; PAS: periodic acid–Schiff; α-SMA: alpha-smooth muscle actin

Histopathological examination of lung tissue revealed that HFD mice lacked eosinophilic inflammation or goblet cell proliferation (Fig. 3A). However, MT staining indicated increased peribronchial and perivascular fibrosis in the HFD group, a condition mitigated by alirocumab or statin administration (Fig. 3A and B). This observation was corroborated by HIS of α-SMA, showing heightened expression in the peribronchial and perivascular regions of HFD mice, which was effectively reduced by either alirocumab (3 and 10 mg/kg) or statin administration (Fig. 3A and C).

To confirm these histological findings, we assessed mRNA expression of collagen 1, collagen 3, and fibronectin in lung homogenates (Fig. 3D–F). The HFD resulted in increased expression of collagen 1 (*P* = 0.008), collagen 3 (*P* = 0.017), and fibronectin (*P* < 0.001) mRNA levels, all of which were consistently diminished by alirocumab (3 and 10 mg/kg) or statin administration.

### PCSK9 inhibition or statin administration suppressed RAS activation in HFD mouse lungs

**Fig. 4:**
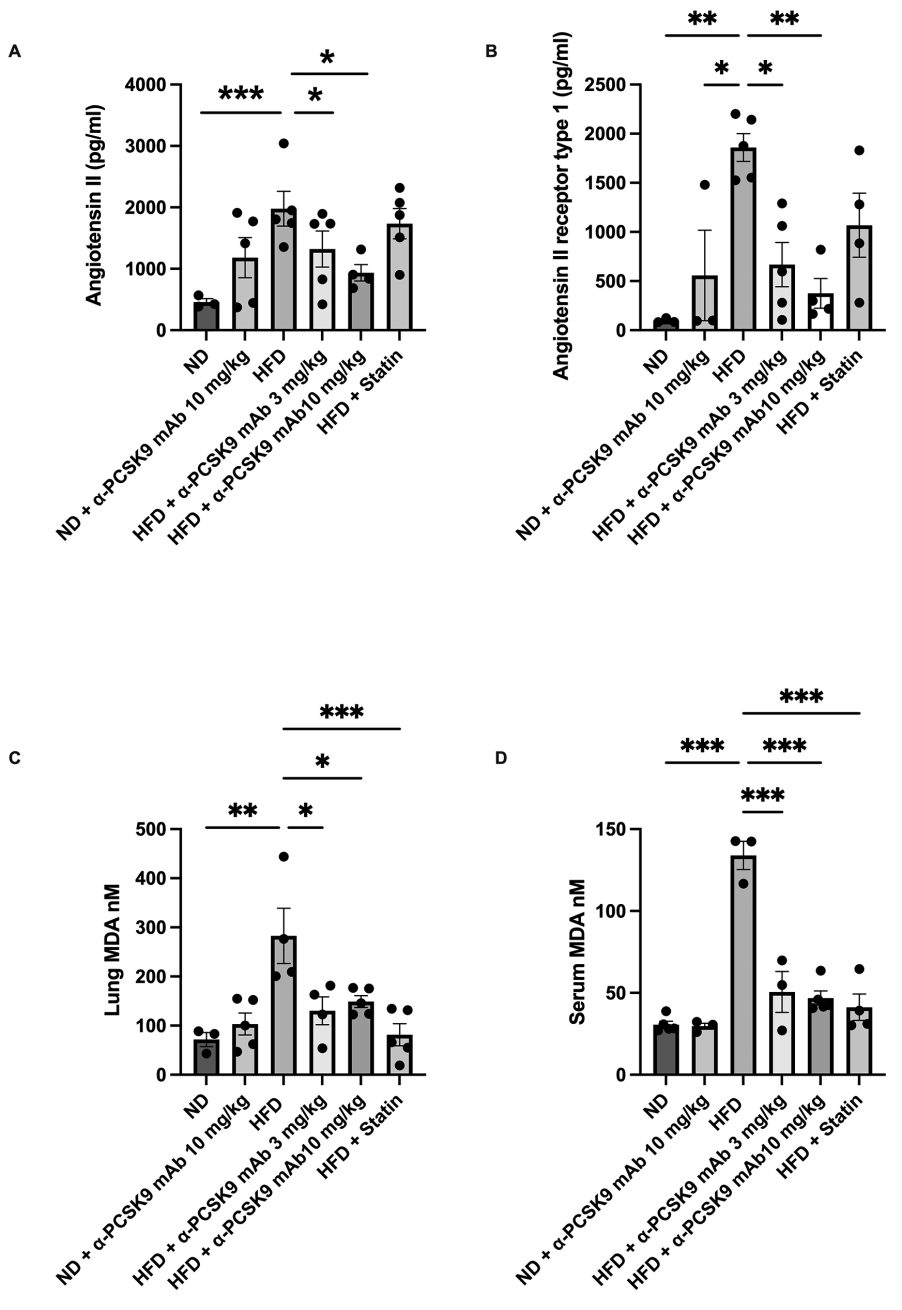
Effects of PCSK9 inhibition or statin administration on RAS activation in HFD mouse lungs. Protein expression of angiotensin II (**A**), angiotensin II receptor type 1 (**B**), and MDA levels (**C**) in lung homogenates and MDA (**D**) in serum were measured by ELISAs. The results are expressed as the mean ± SEM (*n* = 6 per group). Statistical analysis was performed using one-way ANOVA with Bonferroni correction. *: *P* < 0.05, **: *P* < 0.01, and ***: *P* < 0.001. MDA: malondialdehyde

Our subsequent investigation aimed to determine if PCSK9 inhibition or statin administration could impede the activation of RAS activity in HFD mice. Comparatively, the HFD group displayed heightened levels of angiotensin II (*P* = 0.024) and angiotensin II receptor type 1 (*P* = 0.002) compared with those in the ND group. Notably, alirocumab administration (3 and 10 mg/kg) mitigated these elevated expression levels. However, the effect of statin on RAS activity in the HFD group was not as pronounced as that of alirocumab (Fig. 4A and B).

Moreover, the oxidative stress biomarker MDA significantly higher level in both the serum (*P* < 0.001) and lung homogenate (*P* = 0.002) of the HFD group compared to those in the ND group. Interestingly, administration of either alirocumab (3 and 10 mg/kg) or statin significantly reduced MDA levels, both locally and systemically, in the HFD mice (Fig. 4C and D).

### PCSK9 inhibition or statin administration decreased CCK expression and FFA levels in HFD mouse lungs

**Fig. 5:**
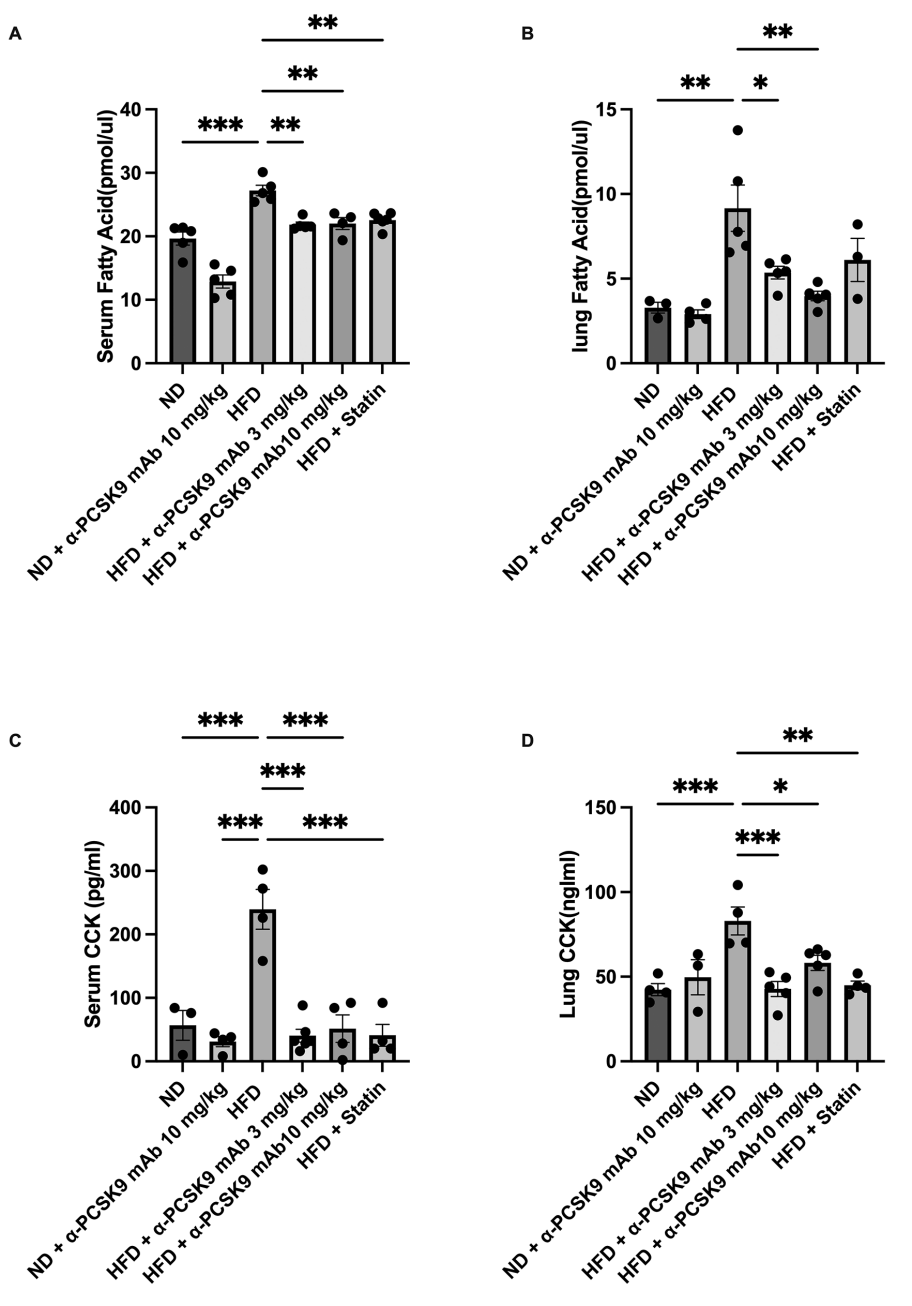
Effects of PCSK9 inhibition or statin administration on CCK expression and FFA levels in HFD mouse lungs. FFA (**A**–**B**) and CCK (**C**–**D**) protein expression in both lungs and serum were measured by ELISAs. The results are expressed as the mean ± SEM (*n* = 6 per group). Statistical analysis was performed using one-way ANOVA with Bonferroni correction. *: *P* < 0.05, **: *P* < 0.01, and ***: *P* < 0.001. FFA: free fatty acid, CCK: cholecystokinin

Obesity correlates with elevated circulating FFAs. In our study, we noted heightened FFAs and CCK levels in serum and lungs of the HFD group compared to those in the ND group. Notably, administration of alirocumab (3 and 10 mg/kg) or statin resulted in significant reduction in FFA and CCK levels in the lungs (Fig. 5A–D).

### PCSK9 inhibition or statin administration decreased NLRP3 inflammasome activity in HFD mouse lungs

**Fig. 6:**
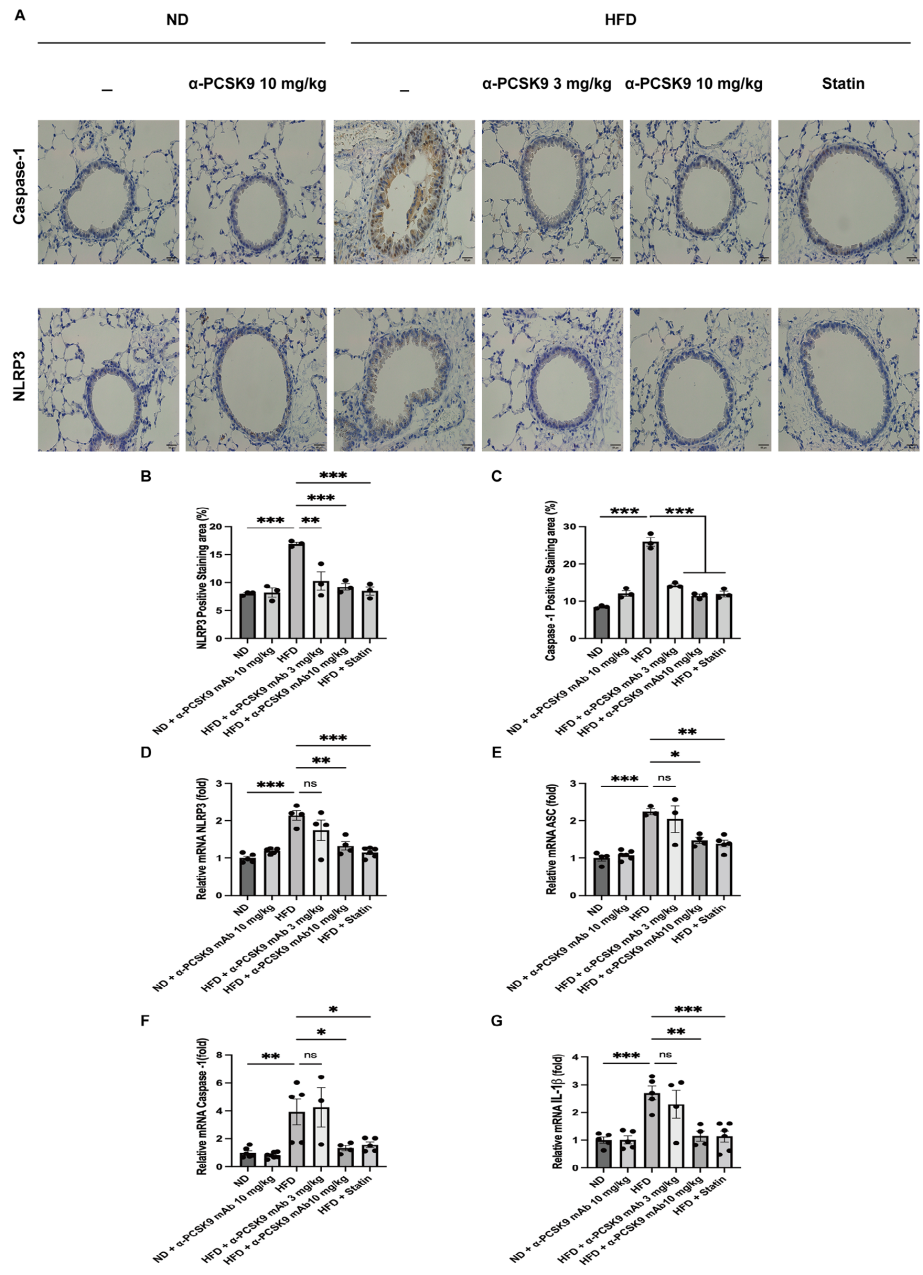
Effects of PCSK9 inhibition or statin administration on NLRP3 activity in HFD mouse lungs. Representative photomicrographs of caspase-1- and NLRP3- positive areas (**A**) in lung sections from mice of the different treatment groups are shown (50×). Quantification of NLRP3- (**B**) and caspase-1-(**C**) positive areas was performed using an image analysis system. Quantitative RT-PCR measurement of NLRP3 (**D**), ASC (**E**), caspase-1 (**F**), and IL-1β (**G**) mRNA expression in the lungs is shown. The results are expressed as the mean ± SEM (*n* = 6 per group). Statistical analysis was performed using one-way ANOVA with Bonferroni correction. *: *P* < 0.05, **: *P* < 0.01, and ***: *P* < 0.001

IHS for NLRP3 and caspase-1 revealed heightened expression in the respiratory epithelium of HFD mice, a phenomenon effectively mitigated by alirocumab (3 and 10 mg/kg) or statin administration (Fig. 6A–C). To further validate these histological findings, we assessed the mRNA expression of NLRP3 in the lungs.

Consistent with the staining results, HFD mice exhibited increased mRNA expression of NLRP3 (*P* < 0.001), caspase-1 (*P* = 0.004), ASC (*P* < 0.001), and IL-1β (*P* < 0.001). Both statin and alirocumab administration were effective in attenuating these elevated level (Fig. 6D–G).

**PCSK9 inhibition or statin administration decreased NLRP3 production of respiratory epithelium and improved lung fibrosis in TGF-β1 overexpressing transgenic mice**.

**Fig. 7:**
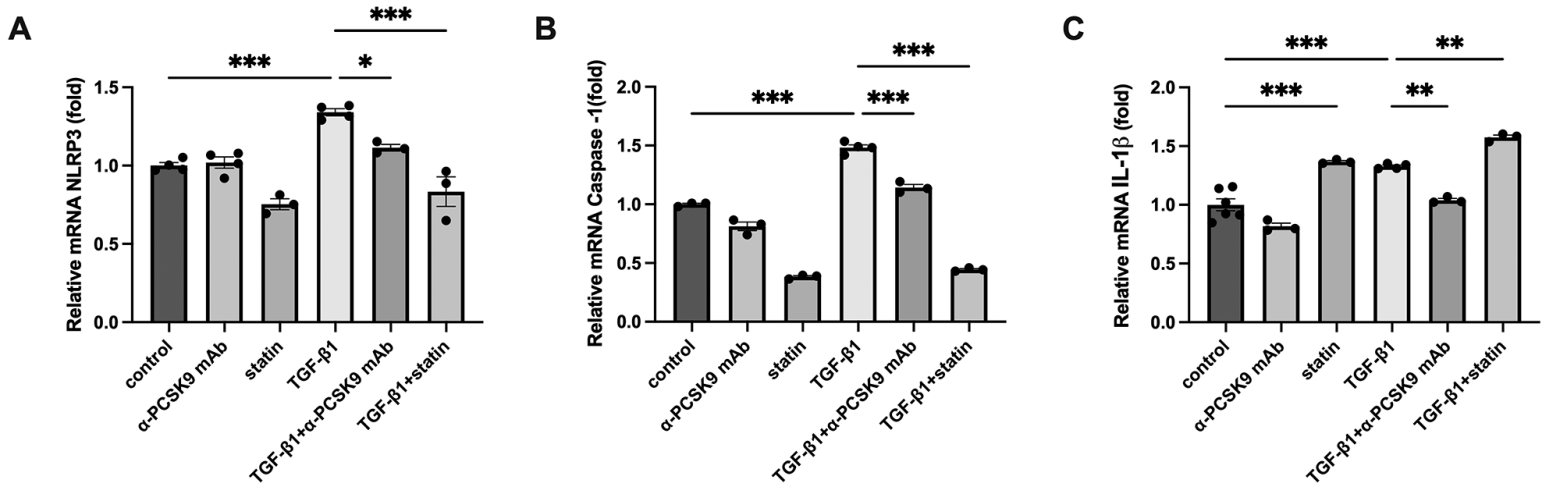
Effects of PCSK9 inhibition or statin administration on the NLRP3 inflammasome activity in respiratory epithelial cells stimulated by TGF-β1. The mRNA expression of NLRP3 (**A**), caspase-1 (**B**) and IL-1β (**C**) are shown. The respiratory epithelium was stimulated with 10 ng/ml of TGF-β1. Statistical analysis was performed using one-way ANOVA with Bonferroni correction. *: *P* < 0.05, **: *P* < 0.01, and ***: *P* < 0.001

Multiple studies have demonstrated that TGF-β1 enhanced NLRP3 expression, subsequently triggering fibrosis in major organs [22, 23]. So, we initiated stimulation of BEAS-2B cells with TGF-β1 gauge the mRNA expression of NLRP3 related mediators. As a result, the mRNA expression of NLRP3 (*P* < 0.001), caspase-1 (*P* < 0.001), and IL-1β (*P* < 0.001) were notably elevated upon TGF-β1 stimulation. Upon administration of alirocumab, there was a reduction in mRNA expression of NLRP3 (*P* = 0.020), caspase 1 (*P* < 0.001), and IL-1β (*P* = 0.001). Statin treatment resulted in a decreased expression of NLRP3 and caspase-1. But, it concurrently led to an increased secretion of IL-1β. (Fig. 7A–C).

**Fig. 8:**
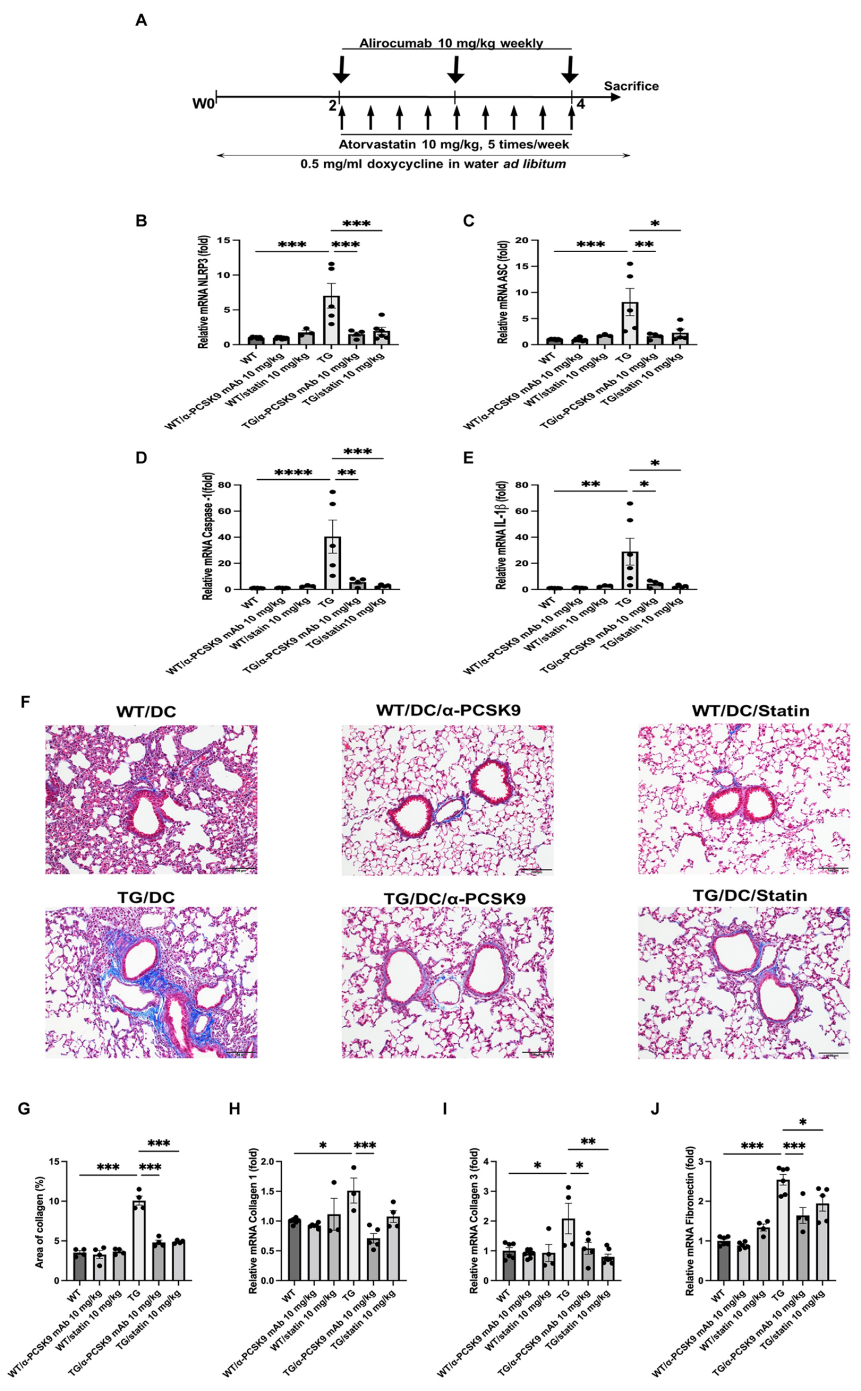
Effects of PCSK9 inhibition or statin administration on TGF-β1 overexpressing transgenic mice. (**A**) Experimental scheme of the TGF-β1 transgenic mouse study. Male and female transgene + mice and transgene − littermates aged 6–8 weeks were fed 0.5 mg/ml doxycycline in water ad libitum for 4 weeks, with intraperitoneal injection of alirocumab (10 mg/kg) weekly and oral administration of atorvastatin (10 mg/kg) five times per week. Quantitative RT-PCR measurement of NLRP3 (**B**), ASC (**C**), caspase-1 (**D**), and IL-1β (**E**) mRNA expression in the lungs is shown. (**F**) MT staining also showed inhibition of peribronchial and perivascular fibrosis by PCSK9 inhibition or statin administration in transgenic mice (original magnification: 100×). (**G**) Quantitative analyses of the fibrosis area were performed using an image analysis system. Quantification of EMT markers in the lungs was performed using mRNA expression of collagen 1 (**H**), collagen 3 (**I**), and fibronectin (**J**). The results are expressed as the mean ± SEM (*n* = 6 per group). Statistical analysis was performed using one-way ANOVA with Bonferroni correction. *: *P* < 0.05, **: *P* < 0.01, and ***: *P* < 0.001. DC: doxycycline; WT: wild-type

Subsequent to this, we investigated whether administration of alirocumab or statin could alleviate NLRP3 activity, ultimately ameliorating lung fibrosis in TGF-β1 overexpressing transgenic mice. TGF-β1 overexpression increased the mRNA levels of NLRP3 (*P* < 0.001), caspase-1 (*P* < 0.001), ASC (*P* < 0.001), and IL-1β (*P* = 0.002), which were notably suppressed upon alirocumab or statin administration (Fig. 8B–E).

MT staining showed an increase in peribronchial and perivascular fibrosis in the TGF-β1 overexpression group, which was mitigated by alirocumab or statin treatment (Fig. 8F and G). Moreover, mRNA of the EMT markers collagen 1, collagen 3, and fibronectin exhibited a decrease following alirocumab or statin treatment (Fig. 8H–J).

## Discussion

Our study underscores the potential advantages of cholesterol-lowering medications such as alirocumab and statins in addressing asthma among patients with obesity by mitigating airway hyperresponsiveness (AHR) and lung fibrosis. In contrast to the typical Th2 high inflammation, a pro-inflammatory environment marked by monocytosis in the lungs may contribute to AHR and fibrosis in asthma patient associated with obesity. Our findings further indicate that these lipid-lowering agents may mitigate this pro-inflammatory state and monocytosis, thus offering beneficial effects for asthma patients with obesity who exhibit inadequate responses to conventional therapies. Further studies are required for the effect of lipid lowering agents using allergic asthma model with HFD induced obesity model.

Obesity often correlates with heightened levels of systemic pro-inflammatory markers like C-reactive protein, TNF-α, TGF-β, leptin, and IL-6 [24, 25], and we have shown that reducing TNF-α and TGF-β1 levels improved AHR and fibrosis in the same HFD-induced obesity model [3, 25]. Factors such as lipopolysaccharide, oxidized LDL, TNF-α, and IL-1β have been noted for inducing PCSK9 secretion in various organs, contributing to conditions like hyperlipidemia, atherosclerosis, diabetes, and hypertension [15]. Studies have highlighted how cholesterol accumulation or cholesterol crystal deposits inside or outside cells, triggers the NLRP3 inflammasome in myeloid cells and macrophages, ultimately playing a pivotal role in generating inflammatory lesions within atherosclerosis plaques [26, 27].

In our HFD-induced model, there was an increase solely in macrophages in the BALF, which was reduced by both lipid-lowering agents. These observations suggest that the anti-inflammatory characteristics of these drugs may involve suppressing NLRP3 activity in macrophages. Our prior research demonstrated that reducing lung macrophages mitigated AHR in a high-fat diet-induced obesity model [25], affirming the critical role of lung macrophages in AHR development.

Numerous clinical studies have highlighted a significant correlation between serum PCSK9 level and pro-inflammatory cytokines like IL-6, IL-1β, TNFα, and hsCRP [28–30]. The activation of TLRs and the NLRP3 inflammasome serves as intermediate steps leading to the production and release of PCSK9 [31]. However, given that obesity is a systemic disease and pro-inflammatory markers were heightened in serum, determining the precise contribution of inflammation - whether systemic, local lung-based, or a blend of both - to lung fibrosis and AHR development remains an open question.

Furthermore, the administration of 10 mg/kg of alirocumab or statin resulted in a modest yet statistically significant reduction in body weight compared to the high-fat diet (HFD) group. It is important to note that the improvement in airway hyperresponsiveness (AHR) observed after treatment with statins or anti-PCSK9 may be attributed to mechanical changes resulting from weight loss. Previous research has established a causal link between obesity-related mechanical alterations, decreased lung function, and AHR, suggesting that this relationship may not solely depend on airway inflammation [32]. However, we posit that the modest weight loss observed in the HFD mice treated with alirocumab or statin might not have been sufficient to induce substantial mechanical changes in this study.

The NLRP3 inflammasome and RAS play crucial roles in the pathogenesis of pulmonary fibrosis [8, 33]. NLRP3 triggers the release of pro-inflammatory cytokines like IL-1β and IL-18. In pulmonary fibrosis, the NLRP3 inflammasome is implicated in perpetuating inflammation within the lungs. The RAS is a hormone system that regulates blood pressure and fluid balance in the body. Beyond its cardiovascular roles, components of the RAS, such as angiotensin II, have been found to be involved in pulmonary fibrosis. Angiotensin II, a key player in the RAS, is known to promote inflammation, fibroblast activation, and collagen deposition in lung tissues, contributing to the development and progression of pulmonary fibrosis.

These two pathways, the NLRP3 inflammasome and the RAS, interact and contribute to a cascade of events leading to chronic inflammation, tissue injury, and ultimately fibrosis [34]. Angiotensin II is known to stimulate the production of ROS, and this ROS production has been linked to the activation of the NLRP3 inflammasome. When NLRP3 is activated by ROS, it initiates a cascade of events leading to the secretion of TGF-β from mouse cardiac fibroblasts [35]. Our earlier investigations revealed that HFD-induced obesity markedly stimulates the RAS and insulin resistance, amplifying the signaling of TGF-β1. These interconnected pathways collectively contributed to the development of fibrosis in the lungs of mice [3]. Consequently, this study suggests that systematically reducing certain biochemical elements by statin or anti-PCSK-9 can attenuate the initiation or stimulation of the inflammasome, potentially improving pulmonary function. These findings align with a recently published study demonstrating that direct inhibition of NLRP3 with MCC950, targeting the NATCH domain, mitigates airway hyperresponsiveness (AHR) and inflammatory cell recruitment in an obesity-induced mode [36]. Understanding and targeting these pathways present promising avenues for developing treatments that could mitigate or even arrest the progression of pulmonary fibrosis. An animal study demonstrated that the elimination of PCSK9 specifically in cardio-myocytes led to the suppression of NLRP3 inflammasome signaling [16]. In our study, the lipid-lowering agents exhibited inhibition of collagen 1, collagen 3, fibronectin mRNA, and SMA protein expression. Consequently, we propose that administering alirocumab or statins might prevent lung fibrosis induced by the overexpression of the TGF-β/Smad signaling pathway.

Remarkably, our in vitro respiratory cell study revealed that TGF-β1 also amplifies NLRP3 expression in downstream pathway, suggesting the autocrine activation of TGF-β1 in NLRP3 pathway in the obesity model, and this phenomenon can also be attenuated through PCSK9 inhibition or statin administration. These findings were supported by our investigation using conditional transgenic TGF-β1 overexpressing mice, which exhibited lung fibrosis and heightened levels of NLRP3, caspase-1, ASC, and IL-1β mRNA, even without concurrent body weight increase. However, these effects were mitigated upon administration of alirocumab or statins. Consequently, we posit that alirocumab or statin administration holds promise in preventing lung fibrosis by suppressing pro-inflammatory cytokines, the RAS, the NLRP3 inflammasome, and subsequently, TGF-β1 signaling, independent of obesity.

Nonetheless, this study was subject to certain limitations, notably its concentration on the NLRP3 inflammasome and downstream signaling pathways in transgenic mice with TGF-β1 overexpression. Future research utilizing the TGF-β overexpressing mouse model should delve deeper into the precise mechanisms through which TGF-β affects both the priming pathway and the secondary activation of the NLRP3 inflammasome.

The elevation of FFAs is a well-documented occurrence in individuals with obesity, attributed to increased FFAs release from adipose tissue or impaired clearance mechanisms [37]. FFAs have the capacity to induce mitochondrial ROS, potentially contributing to inflammation and endothelial dysfunction [38]. Notably, in an HFD-induced obesity model, one study revealed an autocrine stimulatory loop involving CCK-activated, CCKA receptor-mediated airway smooth muscle contraction, a process potentially exacerbated by elevated FFAs [11]. Our observations in HFD mice indicated the increases of both FFAs and CCK in the serum and lungs. Additionally, a research has shown that angiotensin II can upregulate CCK expression at both mRNA and protein levels in cardio-myocytes [39]. Consequently, heightened lung CCK levels in the context of obesity are likely to impact lung function and exacerbate AHR.

Moreover, our findings demonstrated that both alirocumab and statin administration significantly reduced FFAs and subsequently CCK levels in the lungs and serum of HFD mice. This suggests that inhibiting the FFAs-CCK pathway via lipid-lowering agents could represent a promising strategy for treating individuals with asthma and obesity.

In summary, our data indicate that in the HFD-induced obesity model, there are heightened activation of RAS and NLRP3 inflammasome signaling, alongside increased CCK activity. These phenomena are likely due to elevated cytoplasmic LDL-cholesterol and FFAs. These alterations contribute to amplified the expressions of pro-inflammatory cytokines and TGF-β1, culminating in lung fibrosis and AHR. Alirocumab and statins might exert pleiotropic effects in halting these cascades and averting the onset of lung fibrosis and AHR within this obesity model. Further investigations utilizing specific inhibitors of these molecules are imperative to ascertain the precise mechanisms. Lastly, we propose that lipid-lowering agents could represent a viable strategy in treating individuals with asthma with obesity, especially those with inadequate responses to standard asthma medications. Nevertheless, substantiating the clinical efficacy of this approach demands real-world or epidemiological studies involving large sample sizes and long-term randomized clinical trials.

## Conclusions

Our findings suggest that the serum lipid-lowering treatments may alleviate obesity-induced AHR and lung fibrosis through anti-inflammatory responses by inhibition of RAS and NLRP3 inflammasome, and CCK activity. Lipid-lowering strategies may prove beneficial in treating asthma patients with obesity who exhibit poor response to typical asthma medications.

## Data Availability

The datasets in this study are available from the corresponding author on reasonable request.

## Abbreviations

AHR: Airway hyperresponsiveness
PCSK9: Proprotein convertase subtilisin/kexin-9
FFAs: Free fatty acids
RAS: Renin-angiotensin system
HFD: High-fat diet
ROS: Reactive oxygen species
CCK: Cholecystokinin
NLRP3: NOD-, LRR- and pyrin domain-containing protein 3
EMTs: Epithelial transition markers
BALF: Bronchoalveolar lavage fluid
ALT: Alanine aminotransferase
AST: Aspartate aminotransferase
ALP: Alkaline phosphatase
PAS: Periodic Acid-Schiff
H&E: Hematoxylin and eosin
MT: Masson-trichrome
α-SMA: Alpha-smooth muscle actin
HIS: Immunohistochemical staining
TLRs: Toll-like receptors
TGF-β: Transforming growth factor beta
